# Acute Cervical Vagus Nerve Stimulation Modulates Gastric Slow Waves in the Distal Rat Stomach

**DOI:** 10.1111/nmo.70030

**Published:** 2025-03-27

**Authors:** Omkar N. Athavale, Recep Avci, Alys R. Clark, Leo K. Cheng, Peng Du

**Affiliations:** ^1^ Auckland Bioengineering Institute University of Auckland Auckland New Zealand

**Keywords:** gastric electrophysiology, gastric motility, in vivo, pre‐clinical, slow waves, vagus nerve stimulation

## Abstract

**Background:**

Gastric motility is coordinated by bioelectrical events, named slow waves, which propagate across the stomach. Gastric dysrhythmias are associated with disorders of gut‐brain interaction. Electrical cervical vagus nerve stimulation (cVNS) affects gastric contractions, but associated changes in gastric slow waves have not been quantified.

**Methods:**

Three cVNS protocols (low: 0.30 ms, 0.25 mA, 1 Hz; medium: 0.50 ms, 0.50 mA, 5 Hz; high: 1.00 ms, 1.00 mA, 10 Hz) were administered to six rats. Gastric slow waves were concurrently recorded from the serosa of the antrum and distal corpus using flexible electrode arrays. Slow wave amplitude and frequency (mean ± standard deviation) were analyzed with a mixed linear effects model.

**Key Results:**

cVNS had no effect on mean slow wave amplitude (*p* ≥ 0.2208). Slow wave frequency decreased during the high stimulation protocol compared to sham (3.93 ± 0.90 cpm to 3.49 ± 0.54 cpm, *p* = 0.0374, antrum; 3.94 ± 1.04 cpm to 3.15 ± 0.53 cpm, *p* < 0.0001, distal corpus) but returned to sham levels after a recovery period (*p* = 0.9190, antrum; *p* = 0.9995, distal corpus). Ectopic activation of gastric slow waves occurred during cVNS, resulting in a transient effect on gastric slow wave frequency and propagation but not amplitude.

**Conclusions & Inferences:**

Slow wave activity was modified by acute medium and high cVNS stimulation protocols with changes in propagation patterns and mean frequency. Therefore, modified slow wave activity could affect gastric motility function during acute cVNS.

## Graphical abstract

Slow waves were mapped on the rat gastric serosa during acute cervical vagus nerve stimulation with three stimulation protocols. The medium and high cVNS protocols modified slow wave frequency, and slow wave propagation was temporarily disrupted during stimulation in all subjects.

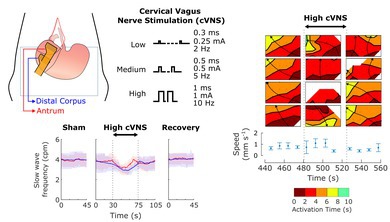

**Summary**

- Rat gastric slow waves were mapped and quantified during cervical vagus nerve stimulation (cVNS).
- Medium (0.50 ms, 0.50 mA, 5 Hz) and high (1.00 ms, 1.00 mA, 10 Hz) cVNS decreased slow wave frequency and temporarily disrupted its propagation.
- Acute high cVNS could have applications in temporarily depressing gastric motility.

## 1 Introduction

The digestion and transport of stomach contents is coordinated by slow waves, which are bioelectrical events that propagate through networks of interstitial cells of Cajal (ICC) and smooth muscle cells (SMC) in the muscular layers of the stomach wall [1, 2]. Slow waves can be generated and propagated spontaneously in the absence of neural input, but it is evident that the parasympathetic nervous system also plays a significant role in regulating the characteristics of slow waves and the ensuing excitation‐contraction coupling [3, 4]. Electrical neuromodulation of peripheral nerves, such as vagus nerve stimulation (VNS), is a potential therapeutic technique for gastric disorders such as gastroparesis, chronic nausea and vomiting, and functional dyspepsia that are associated with abnormal slow wave propagation [2, 5, 6]. However, the extent to which VNS changes the propagation of gastric slow waves remains unclear, especially in commonly used pre‐clinical animal models, such as rats.

The stomach is innervated by neurons of the parasympathetic, sympathetic, and enteric nervous systems, though parasympathetic innervation has a greater influence on contractile function [7, 8]. The rat vagus nerve is comprised of approximately 27% efferent and 73% afferent fibers [9]. However, endings of the fibers do not directly innervate the cells responsible for gastric motility, such as SMC and ICC. Instead, peripheral neuron endings innervate ganglia of the enteric nervous system (ENS), which perform reflex and integrative functions [10]. Enteric neurons innervate effector cells of the stomach responsible for motility [11]. Neural regulation of gastric motility is therefore the integrated effect of both the stomach‐brain axis and the ENS.

Clinical studies of VNS for gastric motility function have used non‐invasive stimulation methods such as trans‐auricular vagus nerve stimulation (taVNS) [12, 13] or transcutaneous cervical VNS [14]. These studies showed that VNS reduced the negative effects of induced bloating in healthy participants [12] and reduced symptoms of gastric dysfunction in patients with existing conditions [13, 14]. These results indicate that VNS is a potential therapy for gastric dysfunction. However, the underlying physiology for these outcomes and the role of gastric slow waves in the overall response remain incompletely understood.

Pre‐clinical experimental studies have shown that VNS can alter feeding behavior [15], contraction frequency, contraction amplitude [16], and gastric emptying [17, 18]. Using magnetic resonance imaging (MRI), Lu et al. [16] also demonstrated a correlation between cVNS intensity and antral motility for stimulation at 5 or 10 Hz, with antral motility being depressed overall. Furthermore, taVNS “normalized” slow waves in a separate study showing a “frequency‐switch” in the behavior of gastric slow waves possibly due to the parameter‐dependent response of different fibers [19]. Abdominal VNS, which has the benefit of reducing off‐target effects on breathing and cardiac function, resulted in a change in the frequency of gastric electrophysiological activity in ferret stomachs [20]. Due to the practicality of transcutaneous stimulation, an understanding of how cVNS affects gastric function and electrophysiology is of particular interest.

This motivates the present work that, for the first time, quantifies the spatiotemporal effect of cervical VNS (cVNS) on rat gastric slow waves in vivo. The objective of the present study was to determine the effect of cVNS on gastric slow waves. This was achieved by measuring slow waves from the gastric serosa with concurrent acute cVNS by a cuff electrode. The high‐resolution extracellular mapping technique [21] used in the present study to measure slow waves was recently validated by simultaneous intracellular measurement in the presence of movement‐inhibiting drugs [22].

We hypothesized that the response of gastric slow waves to cVNS would be parameter‐dependent, and that the antrum would be more sensitive to stimulation than the distal corpus. Repeated measures and a mixed effects linear model were used to reduce the effects of individual variations to increase the robustness of analyses. The outcomes of this investigation will clarify the underlying electrophysiological responses to acute cVNS that could result in modified gastric function. Therapeutic applications of cVNS will benefit from this understanding since disrupted gastric slow waves have been implicated in some functional gastric disorders [2].

## 2 Methods and Materials

### 2.1 Surgical Methods

Male Wistar rats (*n* = 6; 282–312 g; 7–10 weeks old) were obtained from the University of Auckland colony where they were housed in a 12 h/12 h light–dark cycle with food and water available ad libitum. Experiments were approved by the University of Auckland Animal Ethics Committee (approval #23005). All studies started at 09:30, with isoflurane anesthesia induced (4%–5% via induction box) and maintained (2%–3% via nose cone) throughout all procedures. Heart rate, breathing rate, and core temperature were monitored throughout the experiment. Body temperature was maintained using a heated water mat throughout all experiments and a heat lamp, placed approximately 0.5 m away from the animal when required.

**FIGURE 1:**
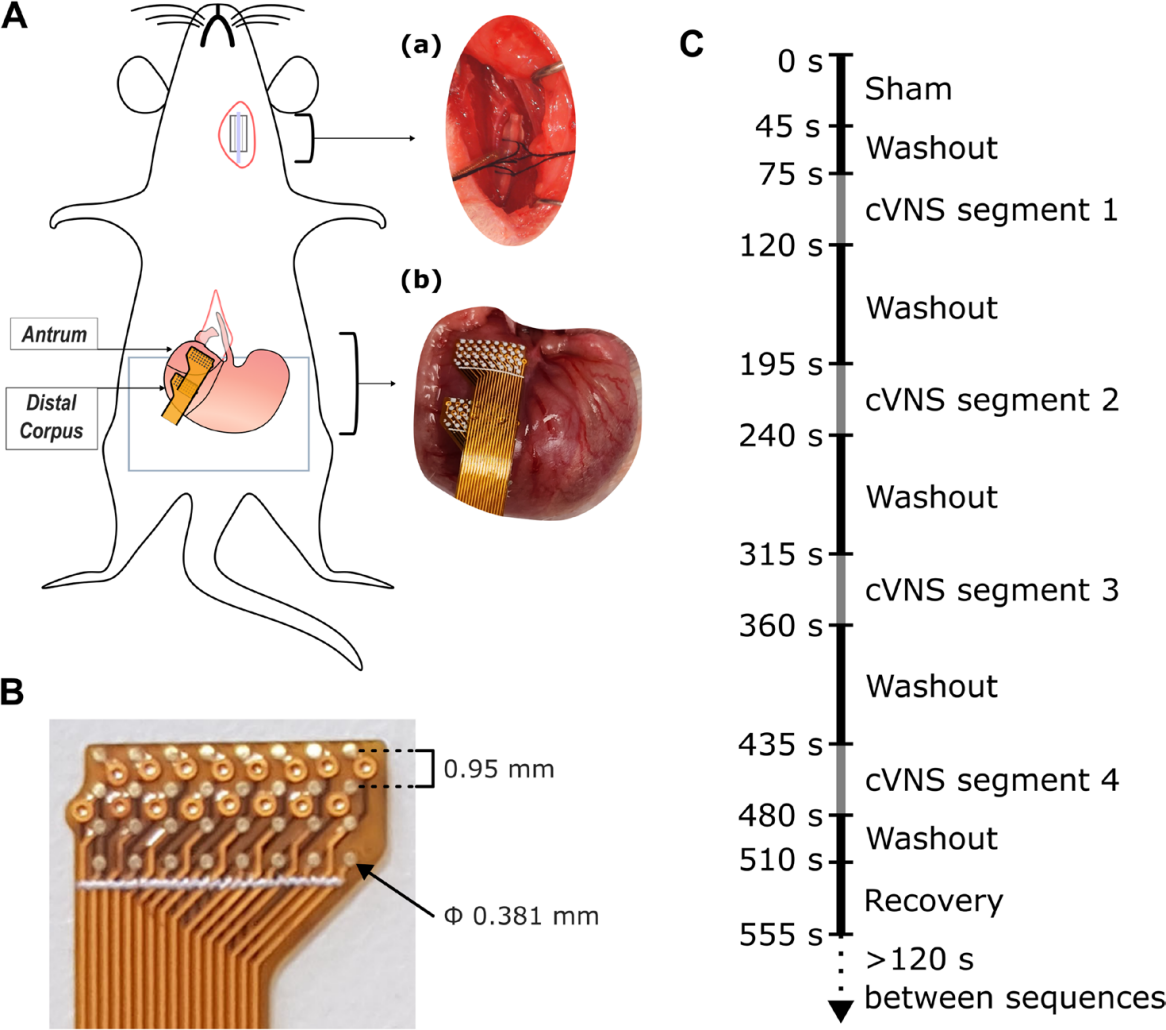
Gastric slow waves were recorded from rats implanted with nerve cuff electrodes on the left cervical vagus nerve. (A) The location of incisions and placement of stimulation and recording electrodes. Photographs of (a) the vagus nerve inside the nerve cuff stimulation electrode, and (b) indicative location of multi‐electrode recording arrays on the ventral gastric serosa. (B) The flexible printed circuit board multi‐electrode arrays used to measure gastric slow waves. Gold‐plated electrodes were 0.381 mm in diameter and were arranged in a 4 × 8 grid with 0.95 mm spacing. (C) The timing of sham, stimulation, and washout segments during each sequence. In each animal, six such sequences were conducted with the order of stimulation protocols varied systematically across the four cVNS segments as tabulated in Supporting Information—Appendix S2.

The nerve cuff electrode was implanted via a subcutaneous incision on the left side of the neck. It had two platinum‐coated ring contacts spaced 1 mm apart, embedded in a silicone cuff with an inner diameter of 0.5 mm (NC‐0.5‐100P‐1.0‐3.0‐00‐300TE‐Ban, MicroProbes Inc., Gaithersburg, MD, USA). The carotid sheath was exposed by blunt dissection, and the left cervical vagus nerve was gently separated (Figure 1A(a)). The cuff electrode was secured in place using suture threads, and leads were exteriorized through the cervical incision, which was sutured shut for the remainder of the experiment.

A midline abdominal incision was then made, through which the stomach was exteriorized, and recordings were taken from the ventral gastric serosa using previously detailed methods [22]. Multi‐electrode arrays constructed as flexible printed circuit boards (Figure 1B) were tessellated, coated with a thin layer of conductive electrode paste (Ten20, Weaver and Company, Aurora, CO, USA), and placed on the exposed gastric serosa (Figure 1A(b)). These were covered with warm, damp gauze and plastic film to retain moisture. Additional warmed (38°C) saline solution was applied as necessary to prevent drying. Signals were acquired at 512 Hz using an ActiveTwo system (BioSemi Inc., Amsterdam, the Netherlands) and saved to a hard drive for processing. This acquisition system configuration had a DC‐coupled bandwidth of 0–400 Hz. ECG electrodes (BlueSensor N; Ambu, Ballerup, Denmark) were placed on the hindlimbs to serve as an electrical reference.

### 2.2 Stimulation Protocol

To deliver current‐controlled stimulation, the nerve cuff electrodes were connected to a DLS100 stimulus isolator which was controlled by a DS8000 signal generator (World Precision Instruments, Sarasota, FL, USA). Stimulation pulses were synchronized to the gastric recordings using a trigger channel from the DS8000 into the ActiveTwo system. Three cVNS protocols were applied as monophasic square waves with varying pulse width (PW), pulse amplitude (PA), and pulse frequency (PF): low (0.3 ms PW, 0.25 mA PA, 2 Hz PF), medium (0.50 ms PW, 0.50 mA PA, 5 Hz PF), and high (1.00 ms PW, 1.00 mA PA, 10 Hz PF). These protocols were within the range shown to be effective at modifying rat gastric contractions in vivo [16]. Partially selective efferent fiber stimulation has previously been achieved by having a rostral anode, requiring monophasic stimulation [16, 23]. Six sequences of different combinations of the three stimulation protocols were administered in each subject (see Supporting Information—Appendix S2). Each sequence contained a 70 s sham segment, followed by four stimulation segments comprising 45 s stimulation, and a washout period of 75 s between stimulation segments (Figure 1C). The sequence ended with 75 s of no stimulation, the final 45 s of which was considered the recovery segment for analysis. At least 120 s passed between the end of one sequence and the beginning of the next. The final 45 s of which was considered the recovery segment for analysis. The cVNS protocols were sequenced such that for all subjects each protocol was administered eight times, being preceded by each other protocol exactly twice, the first protocol of a sequence exactly twice, and the final protocol of a sequence exactly twice (see Supporting Information—Appendix S2).

### 2.3 Gastric Signal Processing

Initial signal processing of gastric slow waves was conducted in the Gastrointestinal Electrophysiology Mapping Suite (GEMS) [24]. Acquired signals were downsampled from 512 Hz to 30 Hz, baseline drift was removed by subtracting the Gaussian running median (10 s window), then the median signal across all channels in each array was subtracted to attenuate common noise, and remaining high‐frequency artifacts were filtered in the time domain using a Savitzky–Golay filter (2 s window, third order polynomial) [25]. Further signal analysis was performed in MATLAB (v2020b, MathWorks Inc., Natick, MA, USA). A continuous wavelet transform (reverse‐padded, time bandwidth product: 20, frequency passband: 0.6–30 cpm) of the filtered signals was taken. From the continuous wavelet transform, the highest power wavelet frequency was determined. The time‐varying power and frequency of this wavelet were respectively stored as the slow wave amplitude and frequency quantities used in further statistical analysis.

Binary classification of slow wave coordination was conducted by inspecting, in a random order, the measured potential of channels arranged along rows and along columns. Each analysis segment with stimulation was classified as coordinated or uncoordinated. The researcher was blinded to all details of the data, such as stimulation on/off, stimulation protocol, subject number, and time of the recording. Detailed analysis of slow wave propagation was performed by first manually marking approximate slow wave event activation times, then automatically adjusting marks to the nearest local minimum of the time derivative of the extracellular potential within 0.4 s of the mark. Slow waves were then grouped using the REGROUPS algorithm [26], and grouping was manually reviewed. Slow wave velocity and frequency were calculated using GEMS, and activation time contour maps were generated to visualize slow wave propagation.

### 2.4 Heart Rate and Heart‐Rate Variability

Heart rate and heart rate variability (HRV) were used to confirm whether cVNS was successfully applied. To determine HRV, the beat‐to‐beat interval (NN interval) was first calculated from the filtered (Butterworth bandstop filter: 45–55 Hz; Butterworth high pass filter: 4 Hz) monopolar gastric signal trace recorded from an electrode located near the heart. The signal energy was then calculated using the Teager‐Kaiser non‐linear energy operator, and peaks of the energy trace were identified as cardiac events using the “findpeaks” function in MATLAB (minimum prominence: 0.05 mV). Then, the time between events was calculated to give the inter‐beat interval. HRV was calculated as the standard deviation of the inter‐beat interval during the analysis segment. When used for short‐duration recordings, this measure of HRV estimates vagal tone, with a high value indicating high vagal tone [27].

### 2.5 Statistical Analysis

Statistical analysis of this repeated measures experimental design was performed in R (v4.3.3; R Foundation, Vienna, Austria). The calculated time‐varying amplitude and frequency quantities were partitioned for each 45 s analysis segment of sham, cVNS, and recovery (Figure 1). Channels were eliminated from analysis when the difference between the 1st and 99th percentile of the measured potential was greater than 2 mV since this suggested that non‐electrophysiological artifacts were present. Using the slow wave amplitude and frequency time series calculated by the wavelet transform, three measures (minimum, maximum, and mean) were quantified for each 45 s analysis segment (Supporting Information—Appendix S5). Outliers more than 1.5 interquartile ranges away from the upper or lower quartile were removed. Statistical analysis was conducted for each of the six measures separately. Summarized quantities were reported as mean ± one standard deviation.

A mixed linear effects model was fitted using the “nlme” package [28], to investigate which cVNS protocols affected slow wave characteristics and determine if the effect was different between cVNS protocols. Fixed effects were estimated for the cVNS protocol and location factors, and random effects were estimated for the subject factor. The normality of residuals was inspected after fitting. Analysis of variance of the fitted model (ANOVA) was used to determine if the cVNS protocol and location had an effect. Contrasts between cVNS protocols, including sham, were estimated using the least‐squares means of the fitted model (“emmeans” package [29]), with p‐values for pairwise comparisons adjusted using the Tukey method and using α = 0.05. Pairwise comparisons were grouped by cVNS protocol within locations and by location for a given cVNS protocol.

Similarly, heart rate variability and mean heart rate during the 45 s segments were analyzed using a mixed linear effects model. However, for these, no fixed effect for location was included, and pairwise differences were compared between each cVNS protocol only.

## 3 Results

Gastric slow wave recordings were recorded from six subjects, comprising eight repeated measurements with each of the cVNS protocols (low, medium, and high) and six for sham cVNS. Measurements were taken simultaneously from the antrum and distal corpus. A total duration of 333 min of recordings was analyzed (55.5 min per subject). Slow waves were present in all the analyzed time segments, with 74% of antrum and 83% of distal corpus channels utilized. The recordings from the remaining channels were eliminated due to artifacts which could be attributed to movement or poor electrical contact [22]. Slow waves during sham stimulation occurred with an amplitude of 0.193 ± 0.042 mV at a frequency of 3.93 ± 0.90 cpm in the antrum and 0.195 ± 0.086 mV at 3.97 ± 1.04 cpm in the distal corpus. The distribution of random effects due to inter‐subject variability had a standard deviation of 0.032 mV, 0.499 cpm, and 35.3 bpm for the mean amplitude, mean slow wave frequency, and mean heart rate, respectively.

### 3.1 Time‐Course of Changes in Slow Wave Characteristics

**FIGURE 2:**
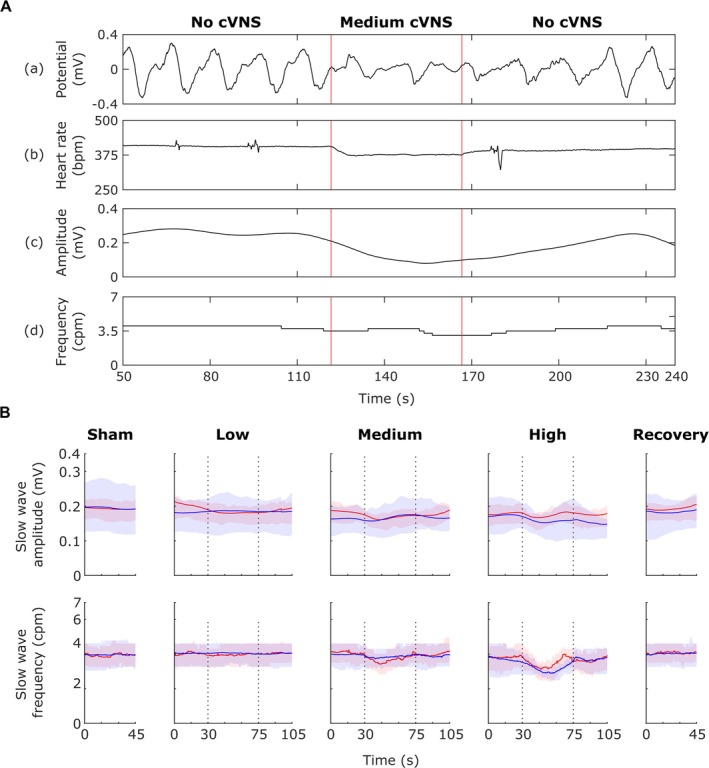
Time course of slow wave characteristics during acute stimulation. (A) A series of traces including a 45 s medium cervical vagus nerve stimulation segment showing the (a) measured extracellular potential, (b) heart rate, (c) calculated slow wave amplitude, and (d) calculated slow wave frequency. (B) Time course of the response of slow wave amplitude and frequency to cVNS. The lines and shaded regions represent the mean and interquartile range for the antrum (red) and distal corpus (blue) across animals (*n* = 6; all male), where the time course for each animal was determined by taking the mean across repeated measurements (6 sham, 8 per protocol, 6 recovery per animal). The sham and recovery panels show the 45 s analysis period, while the low, medium, and high cervical vagus nerve stimulation protocol panels show a 45 s stimulation period between the dotted black vertical lines, along with 30 s prior and after the stimulation period.

An example from a single channel in one subject during the medium cVNS protocol (Figure 2A) shows the gastric slow waves during the sham, medium cVNS protocol, and subsequent washout periods. In this example, the effectiveness of the protocol was confirmed by a corresponding decrease in heart rate from 406 bpm during sham to 378 bpm during stimulation, followed by the eventual recovery to 397 bpm at the end of washout. Similarly, gastric slow wave characteristics also changed during the stimulation period in this example from a single subject (Figure 2A), with a decrease in amplitude from 0.270 mV during sham to 0.080 mV during stimulation, as well as a drop in frequency from 4.02 cpm during baseline to 3.05 cpm during stimulation, with the recovery of both measures by the end of the washout period.

Slow wave frequency and amplitude were modified during cVNS. The averaged time course of slow wave characteristics across analysis segments in all six subjects (Figure 2B) showed that the slow wave frequency was less stable during sham and the low cVNS protocol in the antrum (red lines in Figure 2B) than in the distal corpus (blue lines in Figure 2B). During medium and high cVNS protocols, the antrum and distal corpus exhibited a transient decrease in both slow wave frequency and amplitude just after the onset of stimulation. In the antrum, this transient decrease in slow wave frequency was also followed by a subsequent increase in slow wave frequency, which returned to near sham levels before the cessation of cVNS. These qualitative observations regarding the time‐course were supplemented by statistical comparisons presented in Section 4.3.

### 3.2 Effects on Heart Rate and Heart Rate Variability

**FIGURE 3:**
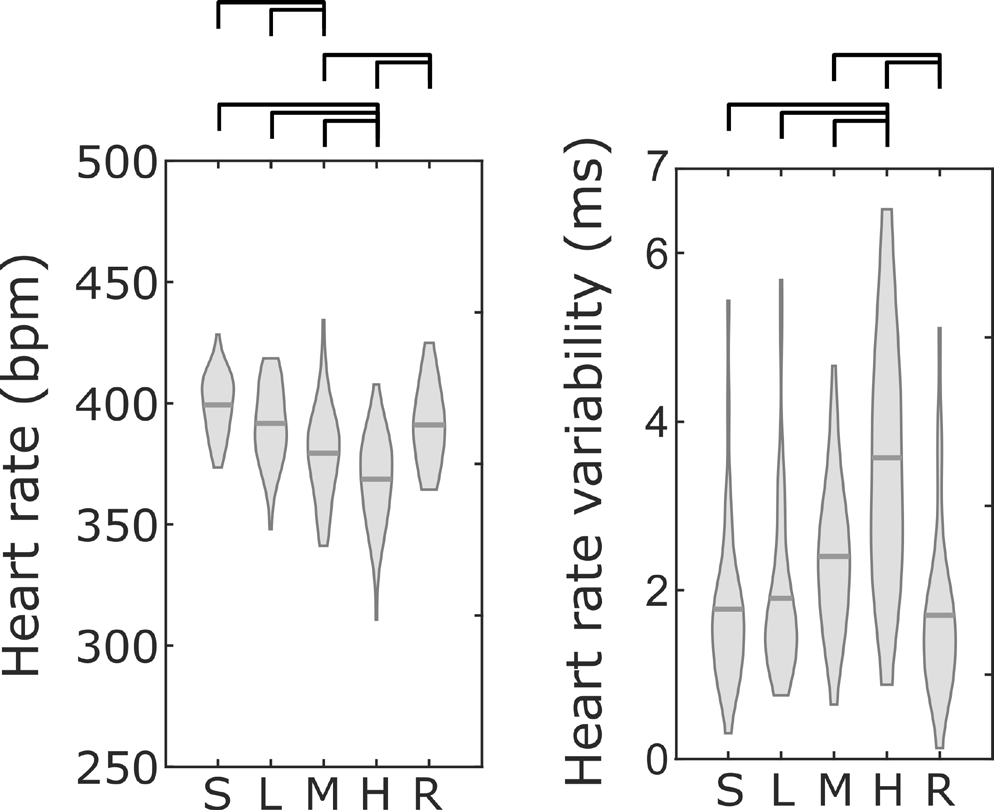
Subject‐adjusted heart rate and heart rate variability during 45 s sham, stimulation, and recovery periods. Heart rate decrease was greater for cVNS protocols having higher charge delivery. Heart rate recovered to the sham level during the recovery segment after a washout period of 30 s. Heart rate variability increased during cVNS and decreased to the sham level during the recovery segment. Violin plots are generated from data with estimated random effects subtracted for subject‐adjustment. The mean is indicated by a horizontal line. Braces indicate the statistically significant pairwise comparisons with Tukey adjustment (*p* < 0.05) between each cervical vagus nerve stimulation protocol from the linear mixed effects statistical analysis. Data were measured from 6 male rats, with 6 sham, 8 low, 8 medium, 8 high, and 6 recovery data per subject. H, High; L, Low; M, Medium; R, Recovery; S, Sham.

The heart rate (Figure 3) was lower than sham (399.37 ± 37.87 bpm) during the medium cVNS protocol (379.35 ± 43.39 bpm; *p* < 0.0001) and the high cVNS protocol (368.64 ± 33.92 bpm; *p* < 0.0001) and recovered to be similar to sham during the recovery period (391.05 ± 42.63 bpm; *p* = 0.2583). Pairwise comparison of HRV between sham and each cVNS protocol and recovery segments showed a significant difference between sham (1.77 ± 1.24 ms) and the high cVNS protocol (4.10 ± 2.37 ms; *p* < 0.0001) and no difference between sham and recovery segments (1.70 ± 1.01 ms; *p* = 0.9987). These results indicate that cVNS increased vagal tone.

### 3.3 Changes in Slow Wave Amplitude and Frequency

**FIGURE 4:**
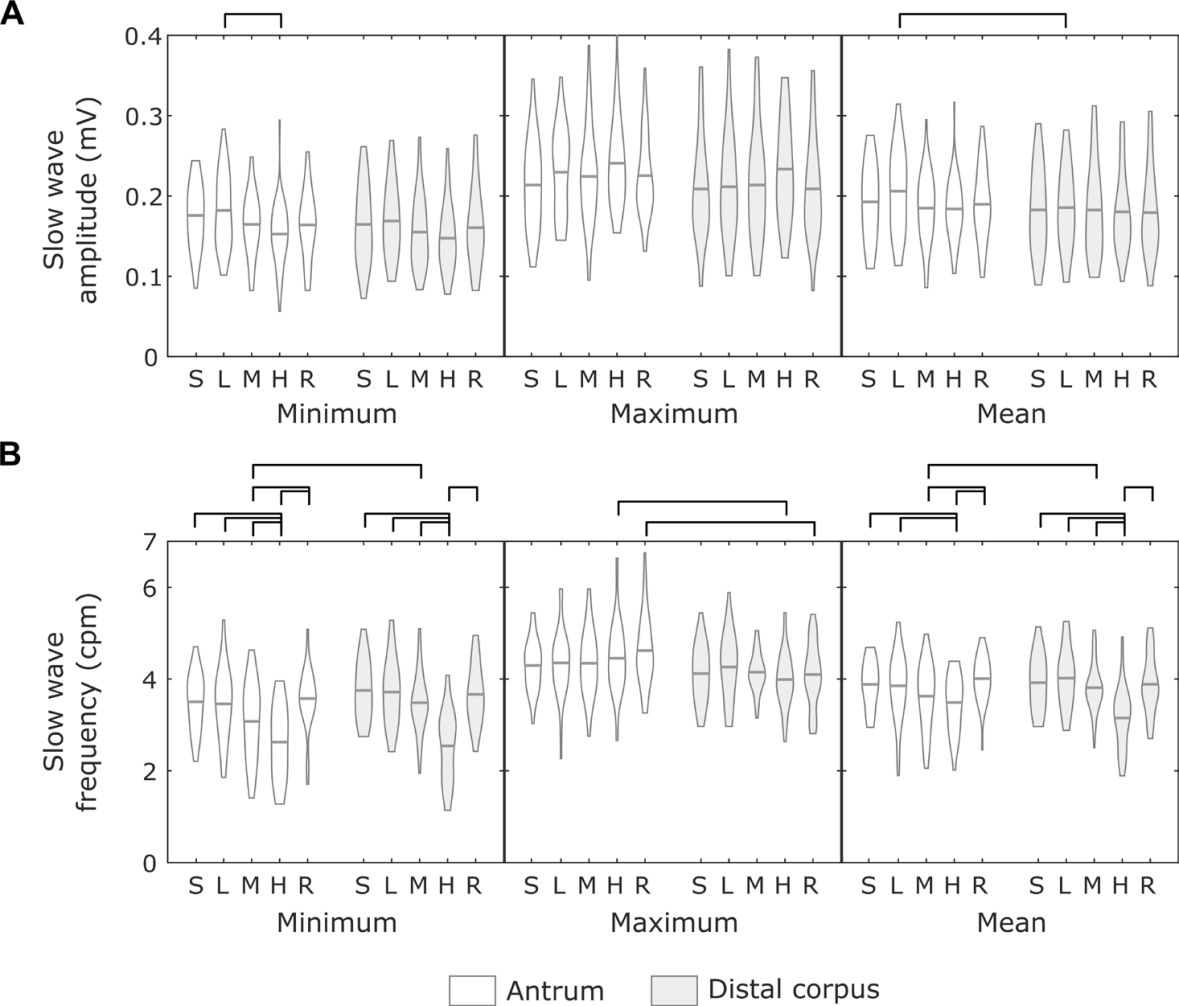
Subject‐adjusted slow wave characteristics during the 45 s sham, stimulation and recovery periods in the antrum and distal corpus. The minimum, maximum, and mean of the (A) amplitude and (B) frequency during each analysis segment are shown. Slow wave frequency decreased during high cVNS in the antrum (white violin plots) and distal corpus (gray violin plots). Slow wave amplitude was not affected by acute cVNS. Violin plots are generated from data with estimated random effects subtracted for subject‐adjustment. The mean is indicated by a horizontal line. Braces indicate the statistically significant pairwise comparisons with Tukey adjustment (*p* < 0.05) between each cervical vagus nerve stimulation protocol or between locations for the same stimulation protocol from the linear mixed effects statistical analysis. Data were measured from 6 male rats, with 6 sham, 8 low, 8 medium, 8 high, and 6 recovery data per subject. H, High; L, Low; M, Medium; R, Recovery; S, Sham.

Statistical analysis of slow wave amplitude (Figure 4A) by ANOVA found that the minimum slow wave amplitude was affected by cVNS protocol (*p* = 0.0012) and location (*p* = 0.0477). However, pairwise comparisons showed that the only statistically significant pairwise difference was between low (0.210 ± 0.060 mV) and high cVNS protocols (0.191 ± 0.049 mV; *p* = 0.0106) in the antrum. As indicated by Figure 4A, there was high variability in the response of slow wave amplitude to cVNS. Therefore, this study did not find statistical evidence that a change in the mean value of the mean, maximum, or minimum slow wave amplitude during the analysis period could be elicited by acute cVNS.

ANOVA of gastric slow wave frequency (Figure 4B) indicated that the cVNS protocol affected the minimum (*p* < 0.0001) and mean (p < 0.0001), but not maximum (*p* = 0.5569) slow wave frequency during cVNS. Location also affected the minimum (*p* = 0.0086) and maximum (*p* < 0.0001) slow wave frequency. Pairwise comparisons showed high cVNS changed the minimum and mean slow wave frequency compared to sham in both the antrum and in the distal corpus. The minimum slow wave frequency in the antrum decreased from 3.56 ± 0.99 cpm to 2.63 ± 0.67 cpm (*p* < 0.0001), while in the distal corpus it decreased from 3.81 ± 1.03 cpm to 2.54 ± 0.56 cpm (*p* < 0.0001). The mean slow wave frequency in the antrum decreased from 3.93 ± 0.90 cpm to 3.49 ± 0.54 cpm (*p* = 0.0374), and from 3.94 ± 1.04 cpm to 3.15 ± 0.53 cpm (*p* < 0.0001) in the distal corpus. The effect of the high cVNS protocol on mean slow wave frequency was also different between the antrum and distal corpus (*p* = 0.0086). In the antrum, the medium cVNS protocol resulted in a lower minimum (3.08 ± 0.82 cpm vs. 3.63 ± 0.93 cpm; *p* = 0.0179) and mean (3.63 ± 0.54 cpm vs. 4.06 ± 0.75 cpm; *p* = 0.0486) slow wave frequency compared to the recovery segment. The effect of the medium cVNS protocol was different in the distal corpus (3.48 ± 0.78 cpm; *p* = 0.0062). A further observation was that the variance, quantified by the aforementioned standard deviation of the slow wave characteristics (Figure 4), was lower for the cVNS protocols with a higher charge delivery. This suggests that the effect of cVNS protocols with a higher charge delivery was more consistent within an animal. The change in variance between stimulation protocols cannot be attributed to changes over time because the application of each protocol was balanced in time by systematically varying the sequence of cVNS protocol application.

In the antrum, medium and high cVNS protocols had different effects on the minimum (*p* = 0.0187) but not the mean (*p* = 0.8154) slow wave frequency. Figure 2B indicates that this may be due to the biphasic response seen during the high cVNS protocol, where slow wave frequency first drops, then increases past the mean sham value within the acute cVNS segment. This biphasic response occurred to a lesser extent in the distal corpus compared to the antrum, explaining the difference between the maximum slow wave frequency during the high cVNS protocol in the antrum and distal corpus (*p* = 0.0010).

Furthermore, all pairwise comparisons for frequency and amplitude quantities in the antrum and distal corpus showed no difference between the sham and recovery segments (*p* > 0.05 for all). The observed effects were transient, and during the recovery segment, the spread of data returned to be similar to sham. Overall, these results show that the acute high cVNS protocol temporarily affected gastric slow wave frequency in the antrum and distal corpus, and that despite the temporary change in slow wave frequency observed during the medium cVNS protocol (Figure 2B) the difference was not statistically significant. Differences in mean slow wave frequency between regions during cVNS suggest that there was a temporary loss of entrainment of slow waves during the medium and high cVNS protocols.

### 3.4 Changes in Slow Wave Propagation

While statistical analysis established that cVNS affected gastric motility, at least in part, through action on slow wave frequency, these changes in frequency were likely associated with the observed disruption of slow wave propagation. Disruption of slow wave propagation during cVNS was identified by classifying the nature of slow wave coordination for each of the 144 stimulation segments compared to the preceding stimulation off segment. The total incidence of changes in slow wave coordination was 51% (74/144). All six subjects exhibited a decrease in coordination, and four of these subjects also exhibited an increase in coordination. The high stimulation protocol had a higher incidence of transitions from coordinated to uncoordinated slow waves (77%) compared to the medium (41%) and low (7%) stimulation protocols. Meanwhile, the incidence of a change from uncoordinated to coordinated slow waves was 57% for both the low and medium stimulation protocols, but 11% for the high stimulation protocol.

**FIGURE 5:**
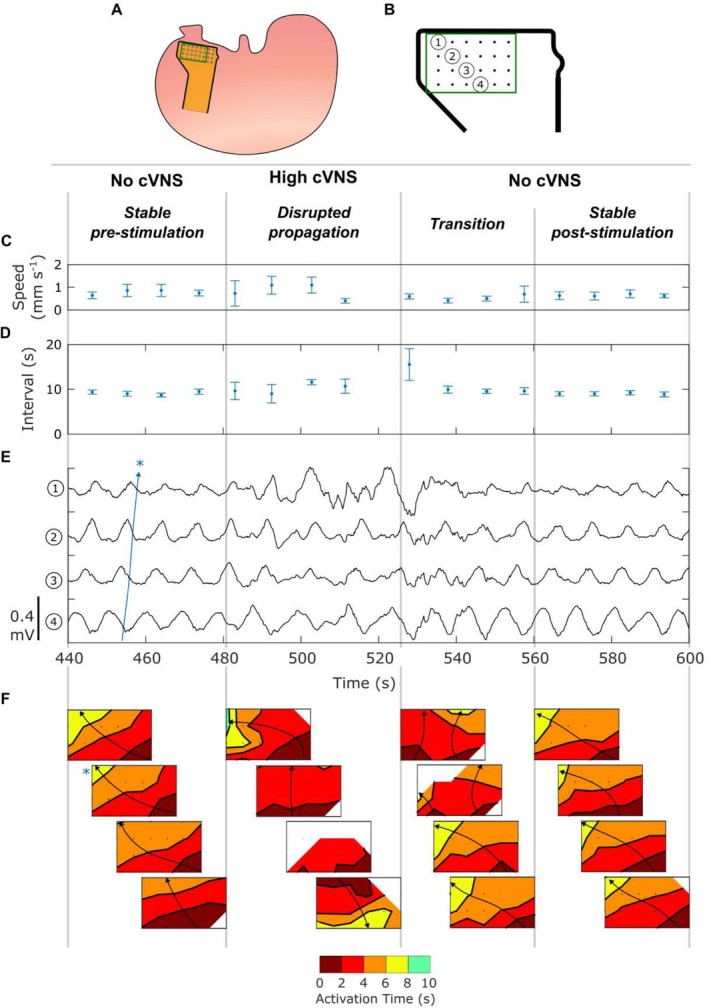
Change in slow wave propagation in the antrum during high cervical vagus nerve stimulation. (A) Position of electrode array on the antrum of the rat stomach. The green box indicates the mapped portion of the array in (F). (B) Locations of electrodes plotted in (E), shown within the green box indicating the area of mapped activation times in (F). Coordinated slow wave propagation was seen before and after stimulation. For each wave the mean ± one standard deviation across channels in the array is shown for (C) slow wave speed and (D) slow wave interval. Both quantities transition back to the pre‐stimulation state following the cessation of cervical vagus nerve stimulation. (E) Traces from a line of electrodes (1.34 mm diagonal spacing) showing the propagation of activity from proximal to distal in the pre‐stimulation, and stable post‐stimulation states. The grouped events of a single propagating wave are marked by a blue arrow to show propagation direction. (F) Maps of isochronal slow wave activation times showing waves for each state. Black arrows indicate the directions of propagation in each map, and a blue star indicates the wave annotated in (E).

Figure 5 demonstrates an example of changing slow wave propagation patterns during stimulation. In this case, during the pre‐stimulation period, stable antegrade propagation towards the pyloric sphincter was observed, with both stable frequency (6.56 ± 0.42 cpm) and speed (0.78 ± 0.23 mm s^−1^) over four cycles. During stimulation, slow wave propagation direction began to change one cycle after the start of stimulation. The speed increased to 1.09 ± 0.40 mm s^−1^ over two consecutive cycles, with a change in the location of the earliest onset of waves within the mapped area of gastric serosa. Notably, the wave morphology also appeared to fractionate in the distal channels near the pylorus (marked channel 1) in the third cycle after the start of stimulation. The direction of propagation was reversed in the fourth cycle after the start of stimulation, though a complete reversal was seen in only two of six subjects. A transition period, lasting approximately 34 s, occurred following the end of stimulation. During this period, slow waves reverted to the antegrade propagation direction with some instability. Finally, stable antegrade propagation re‐emerged four cycles after the end of stimulation. During this period, no fractionated waves were present in any of the channels in the mapping array. Slow wave speed (0.65 ± 0.16 mm s^−1^) and frequency (6.65 ± 0.37 cpm) were comparable to the stable pre‐stimulation period.

## 4 Discussion

The effects of acute cervical vagal nerve stimulation (cVNS) on gastric slow waves were investigated in vivo in anesthetized wild‐type rats. Repeated measurements of extracellular potential from the gastric serosa were taken to assess the response of gastric slow waves to three acute cVNS protocols having low, medium, and high charge delivery. The key outcome of this investigation was that acute cVNS was effective at modulating gastric slow wave frequency, but not slow wave amplitude in the range of applied cVNS protocols.

The amplitude of an extracellular recording is related to the summation of slow waves in nearby tissue. The finding from this study, that slow wave amplitude was not affected by acute cVNS, suggests that nerve stimulation could target pathways other than slow waves to influence gastric motility. Simultaneous measurement of slow waves and contractions in vitro with the chemical application of neurotransmitters has similarly shown that the large decrease in contraction amplitude is not in accordance with the magnitude of slow wave amplitude decrease [30]. However, in vivo studies showed that acute cVNS reduced the amplitude of phasic contractions [16]. Since coordinated slow waves are required for propagating contractions of the stomach [31], it is possible that the disruptive electrophysiological ectopic events observed during acute cVNS play a more influential role in depressing gastric motility function by disrupting the coordination of contractions. While disrupted propagation was observed in all six subjects, retrograde propagation was a specific, coordinated case of the disruption of gastric slow waves observed in two subjects. Further investigation should be conducted to determine whether this coordinated disruption was related to the anatomical distribution of nerves or the specific state of slow wave propagation at the initiation of cVNS.

No overall excitatory changes to slow wave characteristics due to cVNS were observed in this study. Slow wave frequency decreased in response to cVNS. This is consistent with the results for efferent‐biased cVNS reported in Lu et al. [16], wherein minor excitatory responses were seen in the antrum only with pulse amplitudes and widths lower than the low cVNS protocol applied in the present study. Previous studies have suggested that in cases of artificial stimulation, the overall effect of electrical nerve stimulation on gastric or intestinal motility is inhibitory [32, 33, 34].

Previous in vitro studies also showed that VNS resulting in activation of only excitatory ENS neurons can evoke slow waves to induce a “pacemaker shift” in the antrum [35]. Since the in vitro preparation used a small tissue section, the spatial propagation of slow waves was not measured. Observations from the present study demonstrated that not only were ectopic events induced in vivo, but also that the induced slow waves propagated across the distal stomach and disrupted existing antegrade propagation of slow wave activity. This led to the formation of ectopic slow wave pacemakers during the acute cVNS period, which is consistent with the finding that slow wave frequency was different between different regions of the stomach during medium and high cVNS protocols. However, upon cessation of cVNS, the time to recovery of slow waves to their pre‐stimulation state was relatively quick (2–3 cycles) compared to previously reported investigations where recovery occurred to a stable but bradygastric state 43 s after the cessation of taVNS [19], or from direct pacing of the dysrhythmic small intestine where entrainment persisted for approximately 5 cycles but did not necessarily recover to the pre‐stimulation state after this time [36]. In vivo MRI showed the contraction amplitudes recovered in a similarly quick manner after cVNS [16].

This study showed that coordinated slow wave activity resumed after the cessation of stimulation in healthy rats. Further study in disease models would have implications for the clinical application of acute cVNS, possibly indicating the training period of the cVNS treatment regime should be modified depending on the health of the underlying gastric conduction system. A systematic evaluation of the sensitivity of diseased animal models to neural stimulation parameters would improve the clinical translation of VNS in terms of specificity. The identification of safe and effective cVNS parameters using experimental approaches may be supplemented by mathematical models to refine the selection of effective stimulation parameters [37]. The results in this study suggest that in clinical studies where the effects of cVNS are sustained [14], the mechanism of improvement may not be restricted to a change in slow wave activity. Future studies in a chronic setting, with multi‐day recordings of gastric function, will help to better characterize what role slow waves have in the sustained changes in gastric function after VNS. In future studies, a washout period conditional on slow wave characteristics calculated in real time could be implemented. This is especially important if cVNS is to be used to correct extant gastric slow wave dysrhythmias, for example, in diseased animal models. A statistical comparison of the incidence of changes in slow wave coordination was not possible, since the washout period meant that there were few instances where uncoordinated activity was present before the stimulation segment. Such an analysis would be possible with real‐time monitoring of slow wave coordination.

The limitations of the present study were primarily related to experimental techniques. Experiments were conducted under isoflurane anesthesia, which may invoke slow wave dysrhythmias in pigs [38]. Concentrations of isoflurane at anesthetic levels depress neuronal excitability and reduce vagus nerve activity [39, 40]. Isoflurane was selected for this study because it provides a stable depth of anesthesia and has proven to show good response to VNS in pigs [12], but further studies should consider experimental approaches using conscious animals. Moreover, the filling state of the stomach may also result in the transient occurrence of ectopic pacemakers and effects on vagus nerve activity, as shown in previous gastric distention studies [41, 42]. While efforts were made to minimize variation, the stomach filling state was not controlled in this study. While they were appropriately accounted for during analysis, the incidence of non‐electrophysiological artifacts could have been reduced by using more mechanically compliant electrode arrays [43]. Finally, more coverage of the serosal surface would allow better tracking of the exact location of the ectopic region and the extent of the retrograde propagation resulting from it.

Remaining questions concern the long‐term effects of acute cVNS, and how artificial stimulation may have different effects depending on the prior state of slow wave propagation [44]. Some attention must also be paid to the effect of cVNS on electromechanical coupling in the stomach since this is the final driver of gastric motility.

## 5 Conclusion

This study contributes to the understanding of cVNS as an intervention for modulating gastric motility function. Acute cVNS affected slow wave frequency but not amplitude in the anesthetized rat antrum and distal corpus in vivo. These results indicate that the effects of cVNS are transient and that healthy subjects recover quickly from cVNS‐induced disruption. This work shows that acute cVNS could be applied in situations where temporary disruptions to gastric motility are desired, such as a test of gastric function or delay of gastric emptying. Future work should include the investigation of long‐term effects and test the ability of cVNS‐induced disruption to induce natural regulatory processes to correct gastric slow wave dysrhythmias.

## Author Contributions

O.N.A., R.A., A.R.C., L.K.C., and P.D. conceived the research. O.N.A., L.K.C., and P.D. conducted experiments. O.N.A. analyzed data and drafted the manuscript and figures. All authors interpreted the results, critically reviewed, and revised the manuscript. All authors have approved the final manuscript.

## Disclosure

L.K.C. and P.D. hold intellectual property in the field of gastrointestinal motility. L.K.C. and P.D. are directors and shareholders in FlexiMap Ltd. P.D. is a shareholder in Alimetry Ltd. No industry or commercial support was provided for this study.

## Conflicts of Interest

The authors declare no conflicts of interest.

## Supporting information


Appendix S1. Stimulation Parameters

Appendix S2. Stimulation Sequence

Appendix S3. Table of Averages

Appendix S4. Table of P‐values

Appendix S5. Explanatory Note on Data Analysis and Statistical Models

## Data Availability

The data that support the findings of this study are available from the corresponding author upon reasonable request.
