# Supplementary material for: Acute Cervical Vagus Nerve Stimulation Modulates Gastric Slow Waves in the Distal Rat Stomach
Source: Neurogastroenterol Motil. 2025 Mar 27;37(7):e70030. doi: 10.1111/nmo.70030 (PMC12163215; doi:10.1111/nmo.70030)
Supplement: Supplementary file 5 — Appendix S5. Explanatory Note on Data Analysis and Statistical Models [file NMO-37-e70030-s005.pdf]

Explanatory note on data analysis and statistical models

Each 45 s analysis segment was treated as one observation in this data analysis.

The time course of slow wave frequency, slow wave amplitude, and heart rate were calculated and 45 s analysis segments were extracted. For each segment the mean, minimum, and maximum was calculated. An example of the metrics' calculation is shown in Figure S5.1 using the traces from Figure 2 in the main manuscript.

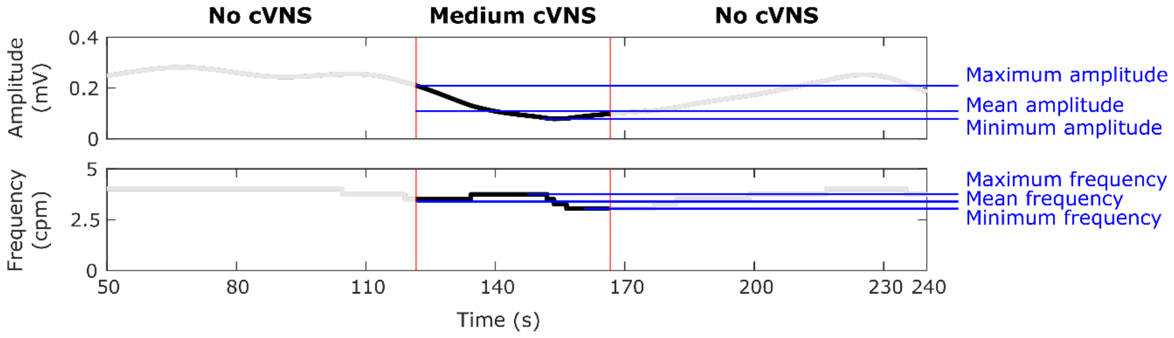

Figure S5.1: Visual representation of metric calculation for amplitude and frequency in an analysis segment where the medium stimulation protocol (0.5 ms; 0.5 mA, 5 Hz) stimulation was applied.

Therefore, each observation had the variables:

- location (antrum/distal corpus)
- protocol (sham, low, medium, high, recovery)
- subject (1, 2, ... 6)
- continuous variables for the metrics
    - minimum frequency
    - maximum frequency
    - mean frequency
    - minimum amplitude
    - maximum amplitude
    - mean amplitude
    - mean sympathovagal balance
    - mean heart rate

The mixed linear effects model apportioned each observation fixed effects for the location and protocol variables, and a random effect for the subject category, as well as a normally distributed random effect for sampling variability. The model was,

$$metric \sim protocol * location + \varepsilon_{subject} + \varepsilon_{iid} \qquad (1)$$

where *metric* is one of the eight continuous variables calculated for each observation.

Using this approach, it was possible to apportion the effect of location and stimulation protocol on each metric within a subject. These results are presented in the manuscript, with reported p-values being those for the respective fixed effects. The random effect due to inter-subject variability was subtracted for Figure 3 and 4 in the main manuscript to highlight the difference in effects due to location and protocol.
